# Transcriptomic analysis of Mesoamerican and Andean *Phaseolus vulgaris* accessions revealed mRNAs and lncRNAs associated with strain selectivity during symbiosis

**DOI:** 10.1038/s41598-022-06566-0

**Published:** 2022-02-16

**Authors:** Joaquín Clúa, Claudio Hernán Rivero, Carla Roda, Carolina Giorgis, Soledad Donna, María Eugenia Zanetti, Flavio Antonio Blanco

**Affiliations:** 1 Instituto de Biotecnología y Biología Molecular, Facultad de Ciencias Exactas, Universidad Nacional de La Plata, CCT-La Plata, CONICET, calle 115 y 49 s/n, CP 1900 La Plata, Argentina; 2 Present Address: Faculté de Biologie et Médecine, Département de Biologie Moléculaire Végétale (DBMV), Lausanne, Switzerland

**Keywords:** Transcriptomics, Plant symbiosis, Rhizobial symbiosis

## Abstract

Legume plants establish a nitrogen-fixing symbiosis with soil bacteria known as rhizobia. Compatibility between legumes and rhizobia is determined at species-specific level, but variations in the outcome of the symbiotic process are also influenced by the capacity of the plant to discriminate and select specific strains that are better partners. We compared the transcriptional response of two genetically diverse accessions of *Phaseolus vulgaris* from Mesoamerica and South Andes to *Rhizobium etli* strains that exhibit variable degrees of symbiotic affinities. Our results indicate that the plant genotype is the major determinant of the transcriptional reprogramming occurring in roots at early stages of the symbiotic interaction. Differentially expressed genes (DEGs) regulated in the Mesoamerican and the Andean accessions in response to specific strains are different, but they belong to the same functional categories. The common and strain-specific transcriptional responses to rhizobia involve distinct transcription factors  and *cis*-elements present in the promoters of DEGs in each accession, showing that diversification and domestication of common bean at different geographic regions influenced the evolution of symbiosis differently in each genetic pool. Quantitative PCR analysis validated our transcriptional datasets, which constitute a valuable source of coding and non-coding candidate genes to further unravel the molecular determinants governing the mechanisms by which plants select bacterial strains that produce a better symbiotic outcome.

## Introduction

Plant roots are surrounded by a vast diversity of microorganisms living in the soil, resulting in the establishment of both benefic and pathogenic interactions. In order to control the number and nature of these interactions, plants have developed sophisticated mechanisms that allow them to recognize, discriminate and respond to microorganism of different kingdoms, species, and even specific genotypic variants (strains, biovars, etc.). These mechanisms are critical to maintain symbiotic relationships in nature, avoiding shifting to pathogenic or parasitic interactions. One of the most ecologically and agronomically important symbioses between plants and microorganisms is the association established between legume plants and nitrogen-fixing bacteria known as rhizobia. This nitrogen fixation process occurs naturally by the interaction with soil bacteria that can live saprophytically in the soil or as endosymbionts inside the root cells. In many legumes, this interaction results in the formation of a new root organ specialized in nitrogen fixation, the nodule, in which bacteria are accommodated within organelle-like structures called symbiosomes^1^. Recent evidence suggests that the root nodule symbiosis originated as a single evolutionary event that was subsequently lost several times in different lineages during the evolution of the orders *Fabales*, *Rosales*, *Curcubitales* and *Fagales*^2,3^. This implies that the adaptative advantage associated with nodulation is relative and probably linked to the fidelity mechanisms between both symbiotic partners. Understanding how these mutualistic symbioses persist in nature would help to unveil the evolutionary history of legume-rhizobium associations. Several models have been proposed to explain how plants are able to control the output of symbiosis, such as partner selection, partner choice or host sanctioning^4–7^. Supporting evidence for each of these mechanisms has been obtained; however, genes involved in the selection of symbiotic partners could have been lost during domestication of cultivated plant species or as a consequence of crop breeding programs. These processes, although have occurred relatively recently in terms of evolutionary times, imposed a severe bottleneck that strongly reduces genetic variability.

The occurrence of partner selection mechanisms constrains the ecological relationships established in agricultural systems, in which a widely used strategy consists in the addition of specific microorganisms to improve crop yields through growth promotion or better usage of soil mineral resources. An example of this principle is the addition of rhizobium strains to alleviate soil nitrogen deficiency. However, strains optimized in nitrogen fixation are usually outcompeted by wild strains present in the soil, which are better competitors but poor nitrogen fixators, thus reducing the effectiveness of the strategy. A better comprehension of the molecular mechanisms underlying this selection by the plant is a necessary step toward improving the design of inoculants that are better competitors in nature.

In common bean (*Phaseolus vulgaris*), partner selection has been associated with the coevolution of both symbionts^8^. This legume species originated in Mesoamerica and then spread and colonized new environments in the south, reaching the Andean region^9^. Geographical isolation gave origin to two centers of genetic diversification (CGD), one in Mesoamerica and one in the South Andes, where parallel evolution resulted in two distinct genetic pools. These pools were subsequently domesticated at each CGD^10^. Remarkably, plants from the Mesoamerican genetic pool retained the capacity to select rhizobial strains that are better competitors and display a better symbiotic outcome^8,11,12^. This capacity was retained in both wild and domesticated accessions^8^, providing a valuable biological system to elucidate the molecular mechanisms and genetic reprogramming underlying this strain preference. The distribution of *Rhizobium etli* -the predominant rhizobia species associated with common bean nodules in America- in Measomerican and Andean soils was investigated using amplified restriction fragment length polymorphism (ARFLP) that characterized strains according to polymorphic alleles of the *nod*C gene^8^. Rhizobia collected from Mesoamerican soils and nodules of wild and domesticated plants from this CGD center harbor mainly the *nod*C-α polymorphic allele, whereas strains carrying the *nod*C-δ allele predominate in Andean soils, as well as in nodules formed in common bean accessions from this CGD (hereafter, strains will be referred to as *nod*C-α and *nod*C-δ). Since coinoculation experiments revealed that Mesoamerican accessions are preferentially nodulated by *nod*C-α strains, it has been suggested that common bean plants have coevolved with *R. etli* strains at the Mesoamerican CGD^8^. A pioneer study using a subtractive hybridization approach identified 43 genes linked to the strain preference displayed by Measoamerican beans^11^. The biological function in the symbiotic strain preference was further characterized for a handful of these genes^13–15^. However, our knowledge of the molecular bases governing the mechanisms by which plants select bacterial strains to establish specific symbiotic associations is still very limited. The majority of the studies in the field have focused on the interaction of a reference plant genotype and a single bacterial strain, leading to the identification of many genes that are essential for the establishment of the nitrogen fixing symbiosis. This approach has been very helpful to dissect the signaling pathway activated by rhizobia^16^, but hindered the detection of genes that influence the outcome of the symbiotic process. Here, we used two domesticated common bean accessions from each CGD and *R. etli* strains carrying either the *nod*C-α or *nod*C-δ allele to elucidate the transcriptional reprogramming of root cells in each of these interactions. This analysis revealed that the plant genotype is the major determinant of transcriptional changes in response to different strains of rhizobia. The regulatory networks governing the transcriptional reprogramming that is common to all rhizobia strains, as well as the specific response to cognate strains, are dictated by distinct families of transcription factors and *cis*-regulatory elements present in the promoters of rhizobia modulated genes, which include both protein coding and non-coding genes. The differentially expressed genes identified in this study are suitable candidates to play a role in the molecular mechanisms underlying strain preference in the nitrogen-fixing symbiosis.

## Results

### Changes in the root transcriptome at early stages of the root nodule symbiosis are mainly determined by the plant genotype

**Figure 1:**
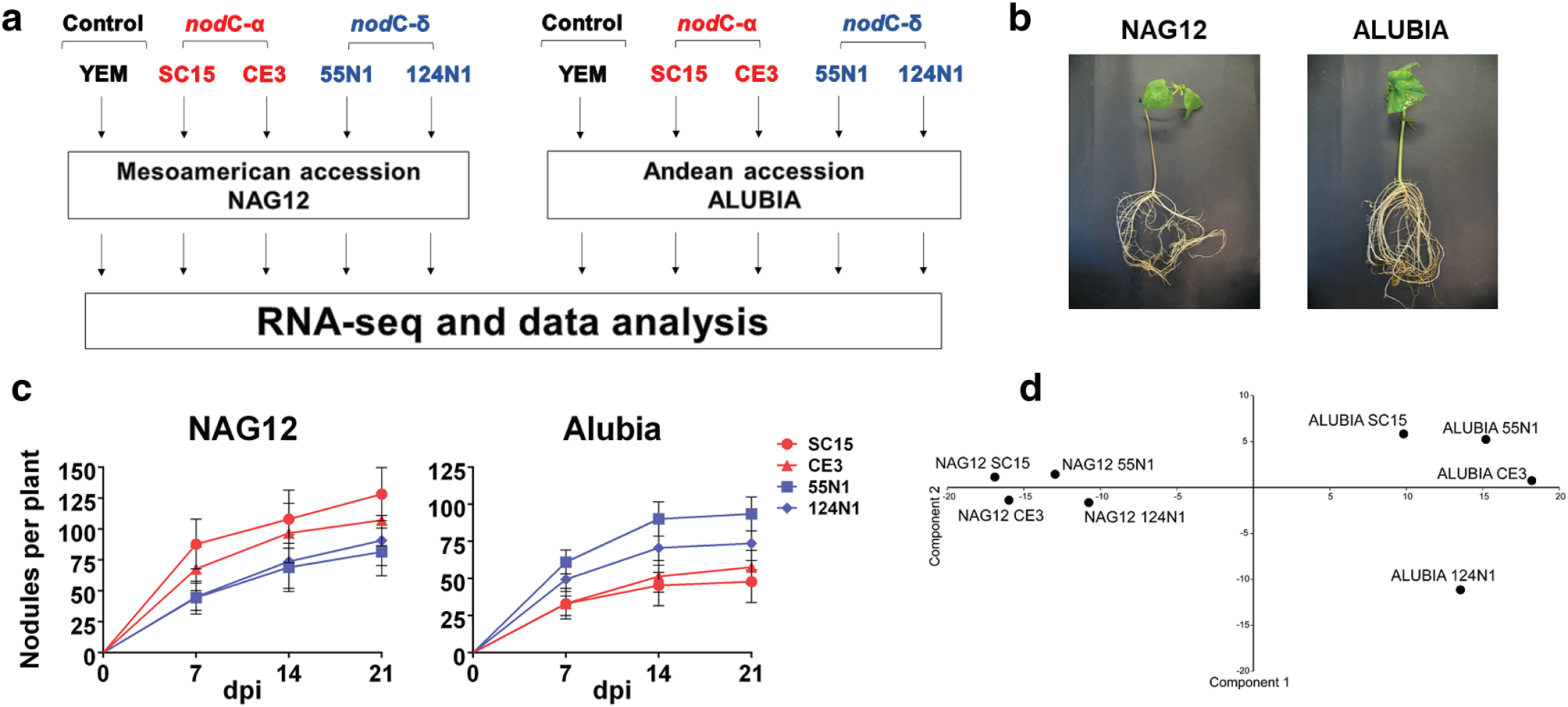
Experimental design to explore strain-specific transcriptomic changes in the *P. vulgaris*–*R. etli* symbiotic interaction. (**a**) Diagram of the experimental design used for RNA-seq studies. *P. vulgaris* NAG12 and Alubia were chosen as representative accessions of the Mesoamerican and Andean genetic pools, respectively. Groups of at least five plants from each accession were treated with growth-culture media (YEM, yeast extract mannitol) as control or inoculated with one of the following *R. etli* strains: SC15 and CE3, carrying a *nod*C-α allele or 55N1 and 124N1 carrying a *nod*C-δ allele. Three biological replicates were obtained for each accession and condition. (**b**) Representative pictures of NAG12 and Alubia accession plants exhibiting comparable root development at 14 days post-inoculation with rhizobia. (**c**) Nodulation kinetics of NAG12 and Alubia accessions inoculated with SC15, CE3, 55N1 or 124N1. The mean number of nodules per plant at 7, 14 and 21 days post inoculation (dpi) is shown (n ≥ 7). Error bars represent the standard error of the mean (SEM). (**d**) Principal component analysis (PCA) plot of NAG12 and Alubia root transcriptomes in response to SC15, CE3, 55N1 or 124N1. Gene expression values used for the analysis were normalized by the corresponding YEM value. The first and second principal components explain 83.0% and 9.7% of the variance, respectively.

To characterize the transcriptional reprogramming of root cells in response to *R. etli* strains with distinct symbiotic outcomes, we selected four different bacterial strains representative of the polymorphism of the *nod*C gene: SC15 and CE3 were chosen as *nod*C-α types, and 55N1 and 124N1 as *nod*C-δ type. One accession from the Mesoamerican (NAG12) and one from the Andean (Alubia) genetic pools of common bean were used as representatives of each CGD (Fig. 1a). Plants from both accessions grow similarly in our experimental conditions, developing a comparable root system (Fig. 1b). In agreement with previous reports^11,12^, the Mesoamerican accession formed more nodules when inoculated with *nod*C-α than *nod*C-δ strains, whereas the inverse nodulation output was observed in the Andean genotype (Fig. 1c). These results verified that each common bean genotype forms a higher number of nodules when plants are inoculated with their cognate strains. To explore early transcriptional changes associated with this different affinity of the symbiotic association, roots of 7-day old plants were inoculated with rhizobia and the tissue of the susceptible zone (defined as the region of actively growing root hairs) was collected 24 h after inoculation, a time in which root hairs are already curled and infection threads start to form^14^. Total RNA from roots inoculated with rhizobia or mock-inoculated with YEM (the media used to grow rhizobia) as control was used to construct and sequence RNA-seq libraries using the Illumina technology. A total of nearly 940 million reads, with an average of more than 31 million reads per library, were obtained (Supplementary Table S1). Reads were aligned to the reference common bean genome obtained from the Andean accession G19833^17^ (*Phaseolus vulgaris* v2.1, DOE-JGI and USDA-NIFA, http://phytozome.jgi.doe.gov/) using TopHat2^18^. On average, 89.1% of the reads obtained from each library aligned to the *P. vulgaris* genome (Supplementary Table S1). Normalized expression values in fragments per kilobase per million reads (FPKM) and transcripts per million (TPM) for each gene in all tested conditions can be found in Supplementary Tables S2 and S3.

The main goal of this study was to elucidate the transcriptomic responses activated by different rhizobium strains in accessions that have diverged since the split of common bean genetic pools into different CGD. To assess the relationship between the transcriptomes of inoculated roots, a principal component analysis (PCA) was performed. The analysis showed that the two main principal components explain 83.0% and 9.7% of the variance, respectively. As shown in Fig. 1d, the datasets were grouped mainly by the plant genotype, suggesting that accessions from each genetic pool of common bean responded to rhizobia by changing the abundance of different subsets of transcripts. Whereas the plant genotype affected similarly samples from both accessions, the rhizobium strain used for inoculation produced more variation in Alubia than in NAG12, suggesting that the response of Andean common beans to individual rhizobial strains is more diverse.

### Transcriptional reprogramming produced by different *R. etli* strains in roots of Mesoamerican and Andean common bean accessions

**Figure 2:**
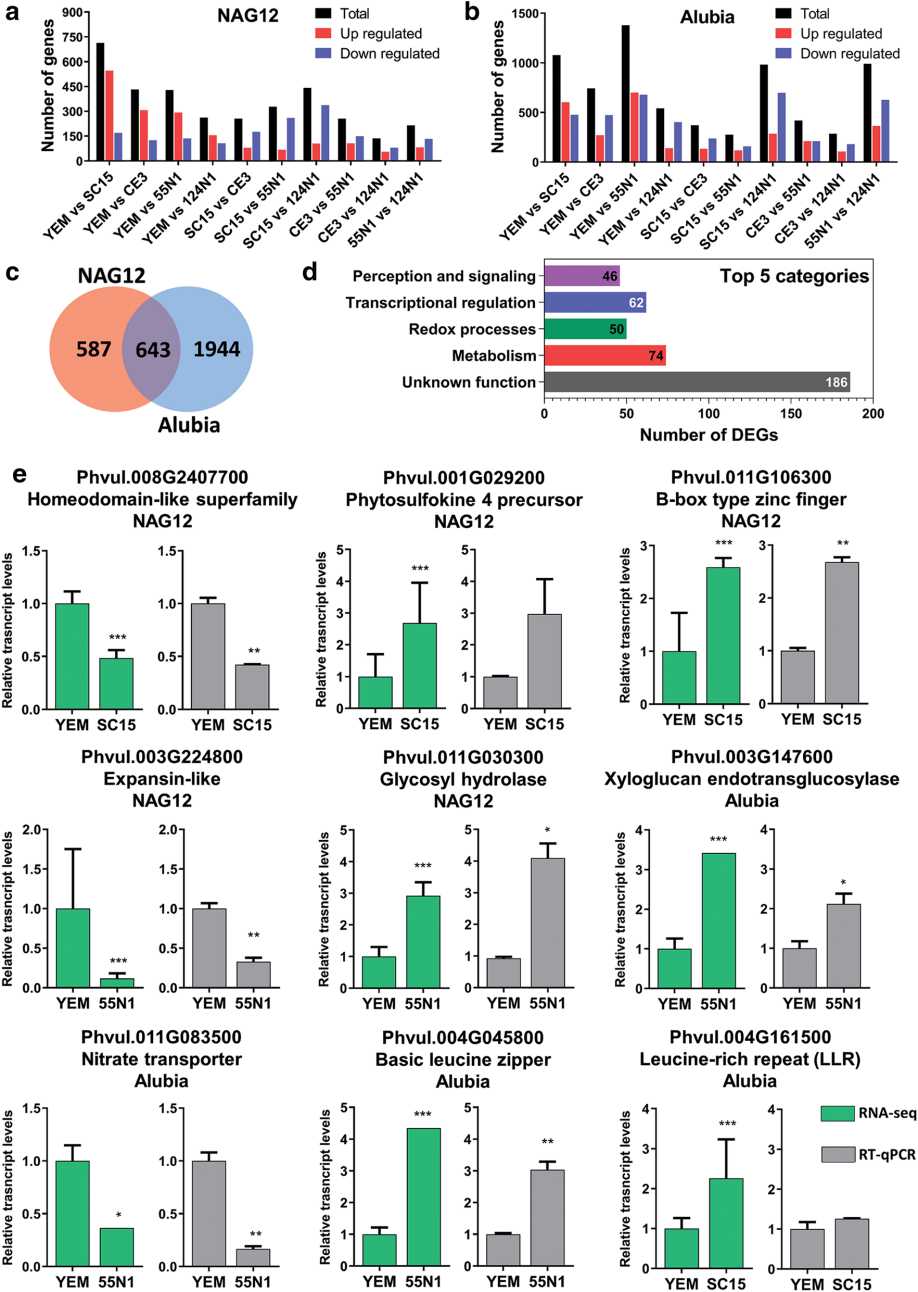
Differentially expressed genes (DEG) detected by RNA-seq and RT-qPCR validation. (**a,b**) Number of total (black bars), up-regulated (red bars) or down-regulated (blue bars) DEGs in each pairwise comparison in NAG12 (**a**) and Alubia (**b**) accessions. (**c**) Venn diagram showing the total number of DEGs in each accession and the number of DEGs common to both accessions. (**d**) Top five functional categories of the DEGs common to NAG12 and Alubia. (**e**) Validation of RNA-seq data by RT-qPCR. The expression of selected DEGs quantified by RNA-seq (green bars) and qRT-PCR (gray bars) is shown. In both cases, expression data is presented as relative to the expression value in the sample treated with YEM, which was set at 1. Bars represent the mean and whiskers the SD. Asterisks indicate significant differences in an unpaired two-tailed Student’s t-test (*p < 0.05; **p < 0.01; ***p < 0.001).

All pairwise comparisons were conducted for each common bean accession. Differentially expressed genes (DEGs) were defined as those that showed a fold change ≥ 2 between two conditions, a p value < 0.05 and a fragment per kilobase per million reads (FPKM) value > 1 in at least one condition. In the Mesoamerican accession, 1230 DEGs were detected in all possible pairwise comparisons, with a maximum number of 714 DEGs (545 up- and 169 down-regulated) between SC15 and YEM inoculated samples (Fig. 2a; Supplementary Table S4). The number of DEGs was higher in Alubia, with a total of 2587 DEGs detected in all possible pairwise comparisons (Fig. 2b; Supplementary Table S5). In this accession, the highest number of DEGs was found in the comparison between 55N1 and YEM (700 up- and 679 down-regulated). A comparison of DEGs revealed a group of 643 genes that responded to rhizobia inoculation independently of the strain in both accessions, representing 52.3% and 24.9% of the total DEGs in NAG12 and Alubia, respectively (Fig. 2c). This group of DEGs included several sentinel genes known to be induced by rhizobia at early stages of the root nodule symbiosis, such as *nodule inception* (*NIN*), *ethylene responsive factor required for nodulation1* (*ERN1*), *early nodulin40* (*ENOD40*), *rhizobial induced peroxidase* (RIP) and *exopolysaccharide receptor 3* (*EPR3*), which were significantly up-regulated in response to either *nod*C-α or *nod*C-δ rhizobia strains in both plant genotypes, thus confirming that roots effectively perceived the presence of rhizobia and activated molecular responses associated to the symbiotic program (Supplementary Tables S4 and S5).

Functional classification of DEGs modulated by rhizobia in both accessions showed that *metabolism*, *redox processes*, *transcriptional regulation* and *perception and signaling* were among the top five categories, which is consistent with the molecular mechanisms triggered during the early stages of the symbiotic interaction (e.g., perception of rhizobia surface molecules by plant receptors, induction of transcription factors of the nodulation signaling pathway and the transient increase of reactive oxygen species upon NF perception) (Fig. 2d). To validate RNA-seq data, we selected a subset of nine DEGs belonging to these functional categories that respond specifically to either *nod*C-α or *nod*C-δ strains in each common bean accession and verified their differential accumulation by reverse transcription-quantitative polymerase chain reaction (RT-qPCR). The RT-qPCR experiments were performed with RNA of root tissue samples from three biological replicates that were completely independent (i.e., experiments performed on different days) from those used in the RNA-seq experiment. The response to inoculation with a particular strain of *R. etli* showed a similar mRNA accumulation pattern in RT-qPCR analyses and RNA-seq data for the five genes selected in the Mesoamerican accession NAG12 and three of the four DEGs selected in Alubia (Fig. 2e). These results support the reliability and robustness of the transcriptomic approach to identify *bona fide* differential transcripts.

### Common and strain-specific DEGs in Mesoamerican and Andean accessions

**Figure 3:**
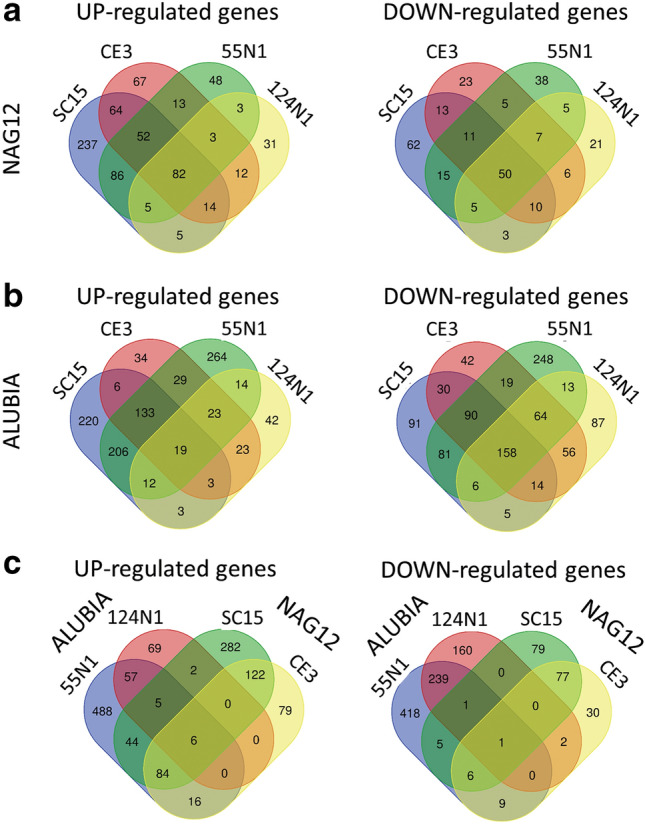
Identification of common and strain-specific differentially expressed genes. Venn diagrams showing the number and overlap of up- and down-regulated DEGs in NAG12 (**a**) and Alubia (**b**) in response to the strains of *R. etli* SC15 and CE3 (*nod*C-α), 55N1 and 124N1 (*nod*C-δ) or in the highly efficient interactions of each common bean accession with their cognate strains (**c**).

Next, we aimed to identify DEGs that respond specifically to each individual strain of *R. etli* in both Mesoamerican and Andean accessions. For this, DEGs were classified according to their fold change in expression level relative to the control sample inoculated with YEM (Fig. 3a,b, Supplementary Table S6). We identified a cohort of DEGs that respond to all tested strains, 132 in the Mesoamerican accession NAG12 (82 up- and 50 down-regulated) and 177 in the Andean accession Alubia (19 up- and 158 down-regulated) (Supplementary Tables S2 and S3). The number of DEGs modulated by *nod*C-α strains (up or down regulated by SC15, CE3 or both) was much higher than that modulated by *nod*C-δ strains (up or down regulated by 55N1, 124N1 or both) in the NAG12 accession (466 vs 146). The opposite was observed in Alubia, where *nod*C-δ strains modulated a higher number of DEGs than *nod*C-α strains (668 vs 423) (Figs. 3a,b). This result indicates that, in both accessions of common bean, cognate *R. etli* triggered a more profound reprogramming of gene expression than allopatric ones, which is consistent with the proposed hypothesis of coevolution of common bean varieties and their sympatric strains at each CGD^8^.

A comparison of transcriptional changes associated with the two interactions that form more nodules in both accessions (i.e., NAG12 with SC15 and CE3 and Alubia with 55N1 and 124N1) revealed overall differences and limited overlap, with only 7 common regulated genes (Fig. 3c). The 6 up-regulated genes encode two genes associated to defense responses: Phvul.010G036100 and Phvul.005G071400, corresponding to a 2-oxoglutarate (2OG) and Fe(II)-dependent oxygenase superfamily protein^19^ and a gamma thionin, a member of the cytochrome P450 family (Phvul.009G061600), a nicotianamine synthase 4 (Phvul.005G052500) and a LEA3 protein (Phvul.007G043000). Except for Phvul.005G071400, all these genes were expressed mainly in 10-day-old roots as compared with other plant tissues according to transcriptomic data available at Phytozome (https://phytozome.jgi.doe.gov/), which is consistent with our biological samples. The only down-regulated DEG detected in all the interactions with higher affinity was Phvul.003G102000, which encodes for a thioredoxin superfamily protein. The putative functions associated to these genes point to a role of redox and stress responses in the establishment of efficient interactions. According to the PCA analysis, the response of Alubia to the *nod*C-δ strain 124N1 is the most dissimilar, as compared with the three other strains. If 124N1 is not considered, the number of genes regulated by the other 3 strains is considerably higher, with 84 up- and 6 down regulated genes. These genes encode several transporters of the ABC, vacuolar and MATE families, cell wall modifiers (expansins, cellulase, pectin lyases), proteins involved in hormone responses and signaling (auxin, cytokinin and gibberellin), cytoskeleton remodeling and two early nodulins (Supplementary Table S6). These genes are part of the common genetic programs associated to early nodule organogenesis and root hair infection. Also, protein kinases, phosphateses and transcription factors can play a role in the perception of the rhizobia molecular determinants and the transcriptional reprogramming that is triggered in the common bean-*R. etli* interaction. However, the relatively small number of genes that are common in the two accessions suggests that the specific responses triggered by sympatric strains are different in plants from each CGD, with only few commonalities in transcriptionally regulated genes.

**Figure 4:**
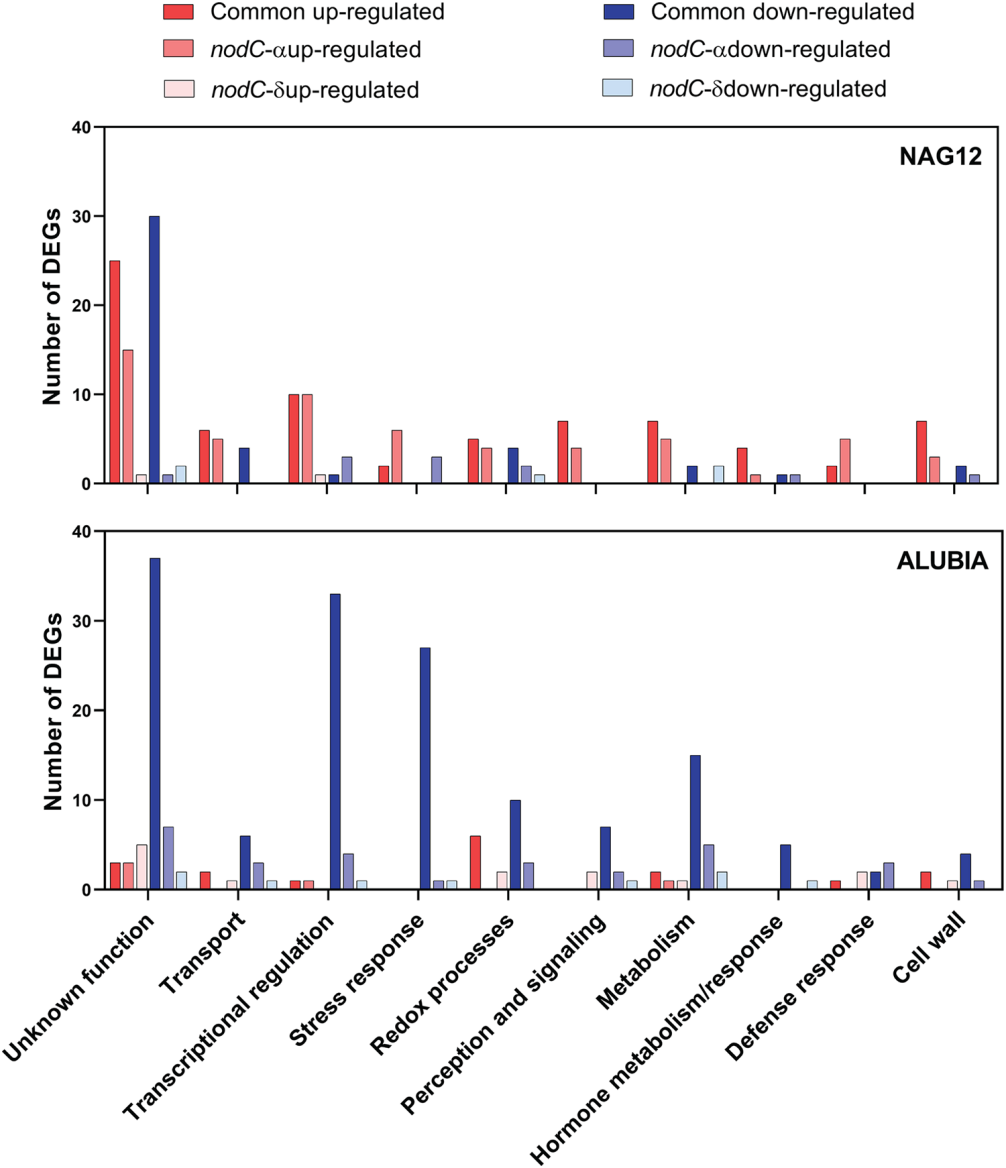
Functional categories of DEGs modulated by *nod*C-α or *nod*C-δ *R. etli* strains in each *P. vulgaris* accession. DEGs that respond to all strains, *nod*C-α (SC15 and CE3) or *nod*C-δ *R. etli* (55N1 and 124N1) strains in NAG12 (upper panel) and Alubia (bottom panel) were manually classified into functional categories using GO terms. Bars represent the number of DEGs up- and down-regulated in each category. The top 10 functional categories are shown.

To further characterize the subset of DEGs that are co-regulated by the four strains and DEGs whose response is specific to *nod*C-α or *nod*C-δ strains in each accession, they were manually classified based on gene ontology (GO) terms (Fig. 4). The first subset contains genes that are part of the common response to rhizobia (such as *NIN*, *ERN* and *RIP*), whereas the other subsets are expected to contain genes that participate in the strain-specific response. Although the identities of the DEGs that respond to all strains were different in both accessions, the main categories were conserved. The function of many up-regulated genes was unknown, whereas other prevalent categories were *transcriptional regulation*, *cell wall remodeling*, *transport* and *perception and signaling*. Most of the down-regulated genes in NAG12 do not have an assigned function. Down-regulated genes in Alubia belong to the category unknown, but in addition, they include transcriptional regulators and stress-induced genes (Fig. 4). This analysis indicates that, despite the genetic differences exhibited in the symbiotic response by the two plant genotypes, similar cellular processes are modulated during early responses leading to nodulation.

To characterize strain-specific responses, we focused our analysis in the DEGs regulated by either the *nod*C-α or *nod*C-δ strains in each accession (Fig. 4). Genes up-regulated in the Mesoamerican accession NAG12 in response to both *nod*C-α strains (SC15 and CE3) belong to similar categories that the general response to the four strains (*unknown*, *transcriptional regulation*, *transport*, and *stress re*s*ponses*). In contrast, *nod*C-δ strains (55N1 and 124N1) did not produce significant changes at the transcript levels in any gene belonging to these functional categories in the Mesoamerican accession. On the other hand, strain-specific responses in Alubia were more complex, involving the same functional categories in up- and down-regulated genes in response to *nod*C-α and *nod*C-δ strains (*unknown*, *transcriptional regulation*, *cell wall* and *metabolism*), but the number of down-regulated genes was higher than the up-regulated ones (Fig. 4). All together, these results suggest that the transcriptional reprogramming includes a common symbiotic response, but also strain-specific changes that affect mainly transcription factors, receptors, and transport proteins.

Transcriptomic changes activated in response to sympatric strains in the Mesoamerican accession included up-regulation of genes encoding cell wall-remodeling enzymes (e.g., pectin lyase and cellulose synthase among others) and cell wall-associated kinases, suggesting that changes in the cell wall at early stages of the symbiotic interaction can influence strain preference in this accession. Also, two genes encoding developmentally regulated transcription factors of the Lateral Organ Boundary Domain (LBD) family, *LBD4* and *LBD11*, were up-regulated in the NAG12 accession in response to *nod*C-α strains (Supplementary Table S6). None of these LBD genes were modulated in the Alubia accession by neither *nodC*-α or *nodC*-δ strains, suggesting that these two specific members of the LBD family might modulate nodule organogenesis and/or development in the Mesoamerican accession in a strain-specific manner. On the other hand, the response of Alubia to *nod*C-δ strains included down-regulation of genes encoding proteins involved in ethylene synthesis, an ACC synthase 1 and an ethylene-forming enzyme, as well as in ethylene signaling response, such as *ethylene insensitive 3* (*EIN3*), three *EIN3-binding F box protein* (*EBF1*) and 5 ethylene response factors, which contrast with the scenario found in NAG12, where the only gene related to ethylene signaling was *EIN3* and it was found to be up-regulated in response to the *nod*C-α strains. This indicates that the Andean, but not the Mesoamerican accession, effectively suppresses the production and signaling of this phytohormone to allow infection of their cognate *R. etli* strains. In addition, the Alubia accession showed down-regulation of 31 genes belonging to the category of stress response, including 12 heat shock proteins (Supplementary Table S6). These differences between both common bean accessions suggest that the responses to sympatric strains are linked to cell wall remodeling and development in NAG12, whereas changes in Alubia are linked to the suppression of stress response and ethylene biosynthesis/signaling.

### Regulation of genes of the nodulation signaling pathways in response to different *R. etli* strains

**Figure 5:**
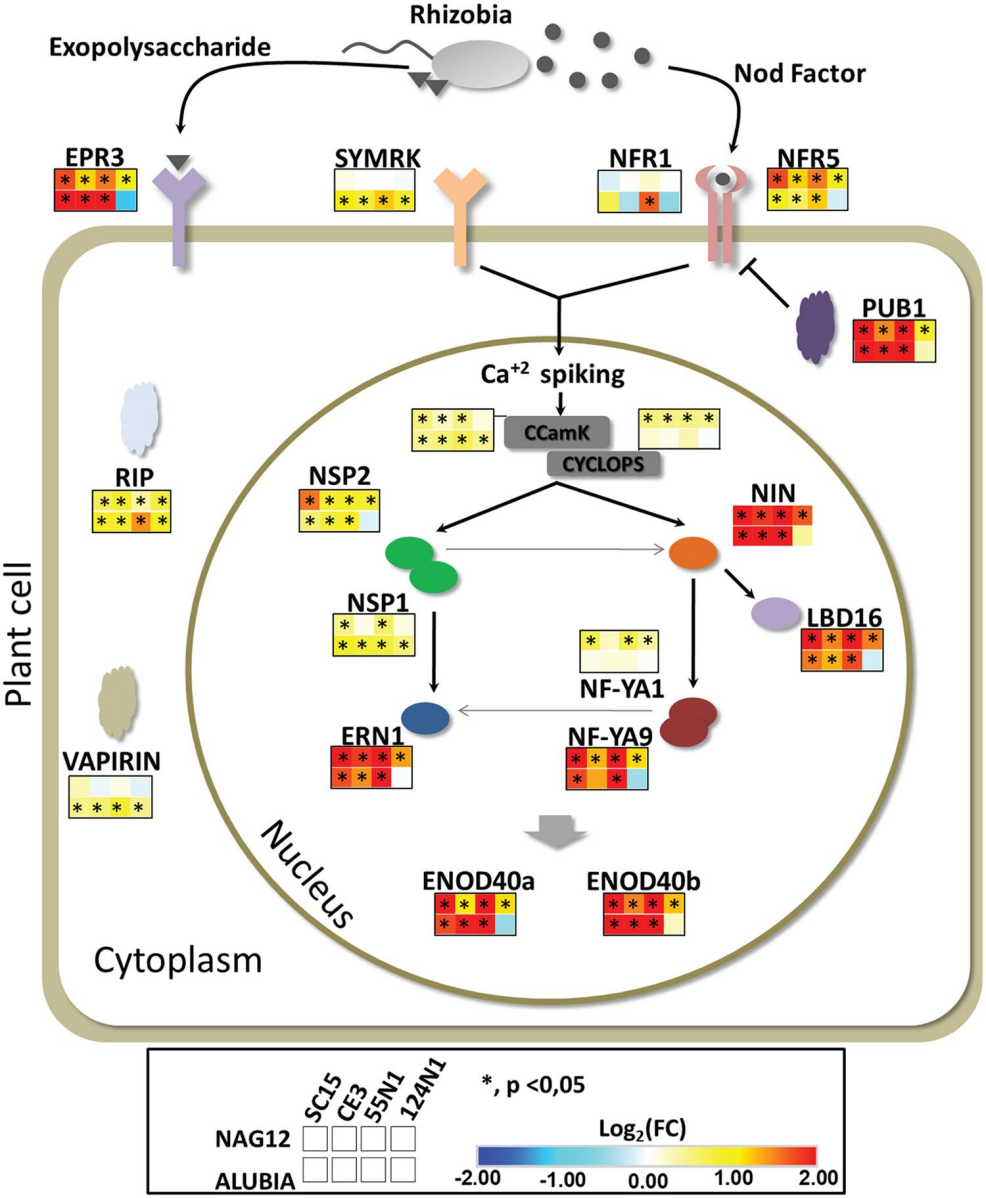
Expression analysis of nodulation signaling pathway components in response to different strains of *R. etli*. The scheme displays the main components of the nodulation signaling pathway triggered by rhizobia inoculation in root cells and their subcellular localization. The Log_2_ fold change (FC) of those genes that were identified as DEGs is shown as a heat map. Squares on the upper and bottom rows show the expression pattern in NAG12 and Alubia, respectively. From left to right *R. etli* strains are SC15 and CE3 (*nod*C-α), 55N1 and 24N1 (*nod*C-δ). The asterisk (*) indicates that the transcript levels in *R. etli* inoculated roots were significantly different from those treated with YEM with a *p* value < 0.05. *EPR3* (Phvul.002G059500), *SYMRK* (Phvul.011G148700), *NFR1* (Phvul.008G211200), *NFR5* (Phvul.002G025500), *RIP* (Phvul.008G249800), *PUB1* (Phvul.008G222100), *NSP2* (Phvul.009G122700), *ERN1* (Phvul.001G111800), *NIN* (Phvul.009G115800), *LBD16* (Phvul.001G159300), *NF-YA9* (Phvul.007G267100), *NF-YA1* (Phvul.001G196800), *ENOD40a* (Phvul.002G064232), *ENOD40b* (Phvul.002G064166), *VAPIRIN* (Phvul.001G113900), *CCaMK* (Phvul.011G186900), *CYCLOPS* (Phvul.002G128600).

Molecular determinants of different rhizobium strains are perceived by specific receptors present in the root cell plasma membrane, triggering signaling pathways that lead to the activation of transcription factors, which are responsible for the transcriptional reprogramming associated with morphogenetic programs. The main molecules secreted by rhizobia and perceived by root cells are the Nod Factors and the exopolysaccharides (EPSs); thus, we evaluated the transcriptional response of a selected group of genes encoding the different proteins of the Nod factor signal transduction pathway and the EPS receptor *EPR3* in both Mesoamerican and Andean accessions upon inoculation with the *nod*C-α and *nod*C- δ strains used in this study. Consistently with previous reports^20–24^, only some of these genes changed their mRNA levels at 24 h post-inoculation (hpi) (Fig. 5, Supplementary Table S7). *NIN*, *ENOD40b*, *Plant U-box protein 1* (*PUB1*), *RIP* and *Ca*^*2*+^*/calmodulin-dependent protein kinase* (*CCaMK*) showed differential expression in both accessions in response to all strains, whereas *EPR3*, *LBD16*, *ENOD40a*, *Nod Factor Receptor 5* (*NFR5*), *ERN1*, *Nuclear Factor YA 9* (*NF-YA9*) and *Nodulation Signaling Pathway 2* (*NSP2*) were induced in both accessions by all the strains except for Alubia in response to 124N1, which showed the weakest transcriptional response for genes of the Nod Factor signaling pathway and *EPR3*. These results indicate that both NAG12 and Alubia accessions are sensing bacterial signals and triggering the signal transduction pathway that will ultimately activate the transcription factors responsible for the reprogramming of root cells for symbiosis. Interestingly, some of the Nod Factor signaling pathway genes were modulated only in one accession, such as the *symbiosis receptor kinase* (*SYMRK*) and *Vapyrin* in Alubia or *Cyclops* and *NF-YA1* in NAG12, at least at 24 hpi (Fig. 5). However, these changes were poorly correlated with the nodulation outcome of the interactions, suggesting that the molecular mechanisms that determine strain-specificity operate independently of these pathways or act at a different regulatory level, which might include post-transcriptional, translational or post-translational events. Another possibility is that these responses and their molecular players are transcriptionally regulated at earlier or later time points of the interaction than that analyzed in this work.

### Transcription factors associated with the strain-specific response

**Figure 6:**
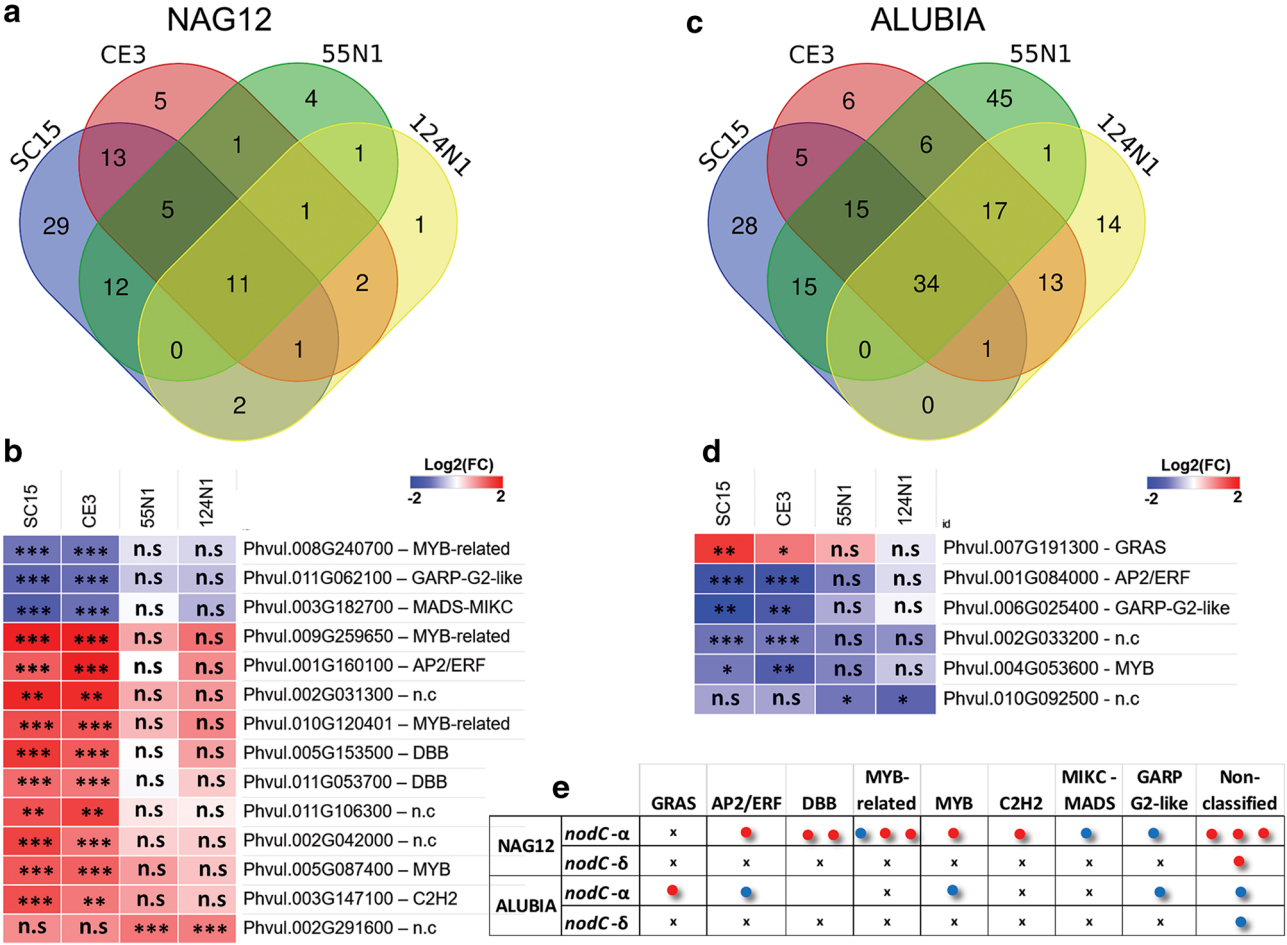
Transcription factors modulated by *R. etli* inoculation in each *P. vulgaris* accession. Venn diagrams representing the number and overlap of DEGs encoding transcription factors in NAG12 (**a**) and Alubia (**c**) after inoculation with the indicated *R. etli* strains. Heatmaps show Log_2_ fold change (FC) in transcript levels of DEGs encoding transcription factors that are differentially accumulated in NAG12 (**b**) and Alubia (**d**) in response to *nod*C-α (SC15 and CE3) or *nod*C-δ (55N1 and 124N1) *R. etli* strains as compared with YEM treated roots. Locus ID and the transcription factor family are indicated on the right. (**e**) Differentially expressed transcription factors classified according to iTAK Database. Classification is indicated in the top row. Each circle represents an up- (red) or down-regulated (blue) transcription factor coding gene. x indicates that there is no DEGs within a particular family of transcription factors in that combination of accession and strains.

DEGs encoding for transcription factors were classified in families using the iTAK database (Plant Transcription factor & Protein Kinase Identifier and Classifier)^25^. In the Mesoamerican accession NAG12, 11 genes encoding transcription factors were modulated in response to the four strains, whereas the *nod*C-α strains SC15 and CE3 affected the mRNA level of 13 genes belonging to different families of transcription factors (Fig. 6a). Among them, mRNAs encoding transcription factors of the Ethylene Response Factor family AP2/ERF, DBB, MYB, MYB related and C2H2 families were up-regulated, whereas mRNAs of genes encoding an MYB-related, a GARP G2 like and a MIKC-MADS family member were down-regulated by *nod*C-α strains (Fig. 6b). In contrast, *nod*C-δ strains 55N1 and 124N1 altered mRNA levels of only one gene encoding a transcription factor in the Mesoamerican accession, which has not been classified in any family. In Alubia, changes in mRNA abundance of 34 genes encoding transcription factors were detected as responsive to both *nod*C-α and δ strains (Fig. 6c), whereas 6 transcription factor coding genes responded specifically to either *nod*C-α or *nod*C-δ strains. Inoculation with the *nod*C-δ strains 55N1 and 124N1 down-regulated the mRNA level of a gene encoding a non-classified transcription factor. On the other hand, *nod*C-α strains SC15 and CE3 up-regulated mRNA levels of a member of the GRAS family and down-regulated mRNA levels of members of the AP2/ERFs, MYB and GARP G2-like families of transcription factors (Fig. 6d). Although in some cases *nod*C-α and *nod*C-δ strains triggered changes in the same families of transcription factors in both plant genotypes, the individual members and responsiveness (up- or down-regulation) to rhizobia strains were distinct (Fig. 6e). In conclusion, changes in the mRNA levels of transcription factors were different in each genotype and these changes were dependent on the specific responses activated by *nod*C-α or *nod*C-δ strains.

To better understand the regulatory transcriptional networks involved in the strain-specific response, we focus our analysis on the promoters of the DEGs in both common bean accessions that were responsive to *nod*C-α and *nod*C-δ strains. The promoters of DEGs were retrieved from the genomic databases generated from the BAT93 Mesoamerican accession of common bean^26^ or the Andean accession G19833^17^. We searched for putative binding sites of transcriptional regulatory proteins (*cis*-elements) in the promoters of the coregulated DEGs and the potential transcription factors involved using the Regulatory gene prediction tool, available at the Plant Transcriptional Regulatory Map. Promoters of DEGs that respond to all strains in NAG12 are enriched in *cis*-elements recognized by transcription factors of the WRKY and HSF (up-regulated), TCP (down-regulated) and AP2/ERF (up- and down-regulated), whereas in Alubia, MYB, AP2/ERF, WRKY and Dof elements are present in down-regulated DEGs (Supplementary Table S8). The number of *cis*-elements in the promoters of DEGs detected in NAG12 as responsive to *nod*C-δ strains was smaller than the number of *cis*-elements present in the promoters of DEGs responsive to *nod*C-α strains, reinforcing the idea that Mesoamerican common beans mount a specific response to strains that lead to select these cognate strains. Comparison between the interaction of NAG12 with *nod*C-α strains and Alubia with *nod*C-δ strains showed that regulatory elements recognized by bHLH, C2H2 and MYB are present in up- and down-regulated DEGs. Some other *cis*-elements are specifically found in one of these interactions, such bZIP (up- and down-regulated), TCP and MIKC-MADS in NAG12 and WRKY (up-regulated) and NAC (down-regulated) in Alubia. This analysis suggests that the geographical isolation of both genetic pools led to the evolution of partially different transcriptional regulatory networks, which contributes to modulate the molecular responses that allow the establishment of a preferential symbiosis in common bean, not only in the general response that governs the execution of the symbiotic program, but also in the strain-specific response that determines the partner selection mechanisms.

### Differentially regulated long non-coding RNAs (lncRNAs)

**Figure 7:**
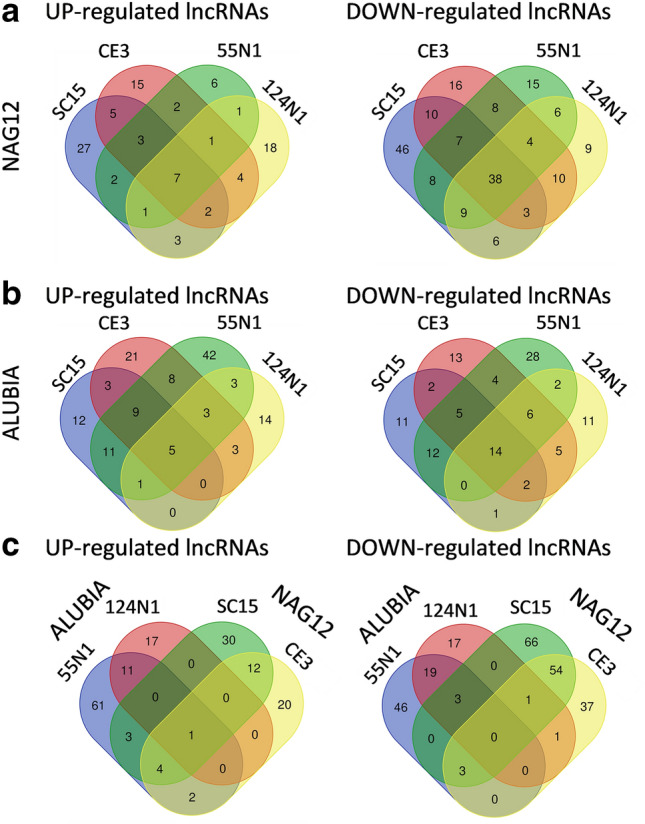
Identification of common and strain-specific DEGs classified as lncRNAs. Venn diagrams showing the number and overlap of up- and down-regulated lncRNAs in NAG12 (**a**), Alubia (**b**) in response to *nod*C-α (SC15 and CE3) or *nod*C-δ (55N1 and 124N1) *R. etli* strains or in the more efficient interactions in each accession (**c**).

Long non-coding RNAs have emerged as critical fine-tune regulators of gene expression that modulate agronomical traits influencing crop productivity^27^. Thus, we focused the analysis of strain-specific responses in DEGs encoding lncRNAs differentially regulated in both common bean accessions by either *nod*C-α or *nod*C-δ strains. We defined lncRNAs as RNAs longer than 200 nucleotides with low- or non-coding potential according to the Coding Potential Calculator 2^28^ and HMMER (see Materials and methods). The number of lncRNAs differentially regulated in response to all strains was 45 and 19 in NAG12 and Alubia, respectively, with a strong predominance of down-regulated lncRNAs (Fig. 7a,b, Supplementary Table S9). LncRNAs were up- or down-regulated in response to *nod*C-α and *nod*C-δ strains in both common bean accessions; however, the identity of these lncRNAs differed between both accessions (Fig. 7c). Only one lncRNA was up-regulated in the interaction of each accession with its sympatric strains, which was extended to 8 when 124N1 was not considered. These results suggest that the modulation of lncRNAs at early times of the symbiotic interaction also has evolved differently in both genetic pools at each GDC. However, there is a small number of lncRNAs that are excellent candidates to study in the context of the strain-specific responses associated to coevolution.

## Discussion

In order to exploit the enormous potential of bacteria that modulate plant growth, it is necessary to understand how coevolution between plants and microorganisms has shaped genomes of both symbiotic partners to control ecological interactions in the soil. In this work, we selected a biological model system based on the divergent evolution of the genetic pools originated in the two CGD of common bean and the specific interaction with strains of rhizobia that are predominant in these soils. Comparison of transcriptomic responses across the different symbiotic associations allowed unveiling the specificity of the transcriptional responses that occur in the plant roots. RT-qPCR analysis verified the reliability of the RNA-seq approach (Fig. 2e), confirming the expression patterns observed in the transcriptomic data in a proportion that was similar to other studies^29,30^. Thus, our transcriptomic approach robustly predicted transcripts that are regulated in response to either *nod*C-α or *nod*C-δ strains in each common bean accession. Besides a general response associated with the general genetic symbiotic program, each common bean accession activated a particular subset of genes in response to different *R. etli* strains, where the genotype of the macrosymbiont was the major determinant factor of the transcriptional response (Fig. 1d). Similar results were obtained in *Medicago truncatula* and soybean^29,31^, suggesting that the strong determination of responses by the plant genotype might be widespread in legume-rhizobia interactions.

### Genes involved in the common and strain-specific responses

Common responses to all strains were considered as part of the general symbiotic programs required for infection and nodule organogenesis, including those genes selected as sentinels in this study and other components of the nod signaling pathway. Some of these responses were conserved between the Mesoamerican and the Andean accessions, whereas others were specifically activated in each variety (Fig. 2), suggesting that the evolution during geographic isolation and domestication has shaped the molecular responses occurring at early stages of the symbiotic process. Genes modulated in both accessions belong to functional categories associated with the cellular response that takes place at the early stages of the interaction, such as perception and signaling and transcriptional regulation, but many of them have not been annotated or do not have an assigned function and need to be characterized using reverse genetics (Fig. 2d).

Previous works have shown that the interaction between Mesoamerican plants with *nod*C-α strains of *R etli* results in higher biomass formation and that these strains are preferentially selected in coinoculation experiments with a mixture of *nod*C-α and *nod*C-δ strains, whereas Andean accession form more numerous nodules with *nod*C-δ strains^8,11,12^. Strain-specific responses were observed in each common bean accession (Figs. 3a,b), but the overlap between regulated genes was limited (Fig. 3c). This result suggests that the evolutionary constrain imposed during domestication has led to the selection of different mechanisms that allow each genetic pool to selectively respond to sympatric strains at each CGD. However, the functional categories involved in both cases are similar, with some specific differences, such as the presence of transport and redox categories in the up-regulated genes in the interaction of NAG12 with *nod*C-α strains, which are not present in the response of Alubia to *nod*C-δ strains (Fig. 4). Interestingly, our results suggest that the response of accessions from each GDC involves different molecular processes. In the Mesoamerican common bean, several cell wall remodeling proteins and two LBD transcription factors are up-regulated in the interaction with *nod*C-α strains (Supplementary Table S6). Early stages of infection require changes at the level of the cell wall that lead to redirectioning the polar growth of the trichoblast to produce the root hair curling and the formation and elongation of the infection thread^1^. LBD transcription factors have been shown to participate in root nodule symbiosis in concert with NF-Y complexes, regulating the reinitiation of cortical cell divisions to form the nodule^32^. These responses can accelerate the infection process and the nodule organogenesis in comparison with other strains present in the soil, allowing a rapid colonization of the root and the preferential occupation of nodules. The DEGs in the Andean cultivar modulated by cognate strains were related to the ethylene biosynthesis and signaling and stress-response (Supplemental Table S6). Ethylene is a phytohormone that negatively modulates infection by rhizobia and nodule formation^33^, suggesting that strain preference in this accession can be mediated by the alleviation of this inhibitory signal. Interestingly, it was shown that ethylene responses in the common bean-*R. etli* symbiosis are modulated by lipopolysacchardes and exopolysaccharides, which are the secondary signals associated to bacterial recognition^34^.

A significant number of DEGs produce lncRNAs (Fig. 7). Whereas several lncRNAs are up- or down-regulated by all strains in each accession (Fig. 7a,b), only one lncRNA was up-regulated in both accession in the interaction with sympatric strains (Fig. 7c). This result is consistent with the highly specific expression pattern associated to genotype, condition and/or tissue reported for lncRNAs, which provide flexible adaptability to gene regulation^27^.

DEGs identified across different samples can reflect differences in the genetic programs triggered in each common bean accession, but also can reflect temporal differences in the transcript stability in each interaction. Broadening this study to other time points, as well as including more common bean accessions, would certainly contribute to a deeper understanding of the genetic differences and the dynamic response between genetic pools from each CGD.

The main determinant of legume-rhizobia compatibility is the NF, which triggers the genetic programs of symbiosis activating the Nod signaling pathway. We observed that some components of this pathway showed a higher mRNA accumulation in response to individual *R. etli* strains, but these changes were poorly correlated with the outcome of the interaction in terms of the number of nodules (Fig. 5). This result is not surprising since the compatibility at a sub-species level might be determined by other signaling molecules produced by rhizobia, such as exopolysaccharides, lipopolysaccharide or proteins secreted by the bacterial type III secretion system^35^.

### Differential genes involved in transcriptional regulation

Signal perception and transduction usually lead to activation of transcription factors, which modulate the activity of the RNA polymerase II to execute the reprogramming of gene expression during development or in response to external stimuli. Our results suggest that the transcriptional reprogramming associated with the general program of nodulation is different than the strain-specific transcriptional response. While the former is activated by Nod Factors, the latter might be dictated by the activation of molecular mechanisms of strain preference and responses triggered by specific determinants of rhizobium identity other than Nod factors. In the Mesoamerican accession, transcription factors of AP2/ERF, DBB, MYB-related, C2H2, MIKC-MADS and GARP2 G2 like families were up- or down-regulated in response to *nod*C-α, whereas *nod*C-δ strains did not produce significant changes in the levels of genes encoding transcription factors (Fig. 6). These transcriptional regulators can contribute to mount a more efficient response when partners with better symbiotic performance are selected in a market scenario. Regulatory elements found in DEGs showed that the transcriptional networks involved in the symbiotic response in NAG12 and Alubia are partially distinct (Supplementary Table S8), suggesting that genetic diversification and subsequent domestication led to the selection and recruitment of different *cis*-elements and cognate transcription factors. More importantly, the results indicate that the molecular mechanisms that participate in the selection or preference for specific strains involve the transcriptional activation of different subsets of genes in each common bean accession. This might explain the differences in the performance of the interaction reflected in the number and size of nodules, the pace of the infection and nodule organogenesis programs and the conditioning of competence in the market scenario of rhizobia within the soils.

## Concluding remarks

The compatibility between legumes and their symbiotic partners is the key to maintain the efficiency of the nitrogen fixing symbiosis. The success of this process relies on the capacity of the plant to select the best available partners in the environment. For this, ancestral species might have evolved molecular mechanisms that allow them to recognize specific signals from rhizobia and trigger the transcriptional activation of genes that favor the infection by these particular strains^7^. The RNA-seq approach described here identified a group of genes that can be used as markers of efficient nodulation. At the same time, it is a first step toward genetically characterize the early specific responses that affect the outcome of the symbiotic process in legumes. Since the success of the strategy of introducing inoculants in cultivated soils is conditioned by the capacity of the plant to select specific strains, this knowledge could be applied in breeding programs attempting to enhance the genetic compatibility between both symbionts.

## Materials and methods

### Biological material and plant inoculation

Seeds from common bean (*Phaseolus vulgaris*) accessions NAG12 and Alubia^36^ were obtained from INTA Salta, Argentina. Growth and inoculation were performed essentially as previously described^14^. *Rhizobium etli* strains used in this study are listed in Table 1 and they were previously described^8,13,15^. Germinated seeds were transferred to acrylic boxes containing Fahraeus media without nitrogen supply. Seedlings were grown in a growth chamber at 26 °C with a 16 h/8 h day/night cycle and 80% humidity. Seven days after transplantation, roots were inoculated with 5 ml of a *R. etli* culture grown in liquid Yeast Extract Mannitol (YEM) media until cultures reached an OD_600_ = 0.8. Nodulation time-courses were obtained by counting nodules at 7, 14 and 21 days after inoculation (dpi) using three biological replicates and a minimum of 5 plants per replicate. For library constructions, a minimum of 5 plants were inoculated with the indicated strains or YEM medium as a control. To avoid biological variations caused by differences in the *R. etli* physiological stage, NAG12 and Alubia plants were inoculated at the same time with the same culture of each bacterial strain. Root tissue of the susceptible zone, which presents actively growing root hairs and is susceptible to rhizobia infection, was collected and frozen in liquid nitrogen 24 h post-inoculation (hpi) with each rhizobial strain. Three biological replicates for NAG12 and Alubia were independently prepared.

**Table 1:** *Rhizobium etli* strains used in this study.

Strain	Genotype^a^	References
SC15	(*nod*C-α)	Aguilar et al.^8^
CE3	(*nod*C-α)	Zanetti et al.^13^
55N1	(*nod*C-δ)	Aguilar et al.^8^
124N1	(*nod*C-δ)	Zanetti et al.^13^
CFNX5 DsRed	(*nod*C-α)	Rípodas et al.^15^

^a^Polymorphic form of the *nod*C gene according to an ARFLP study (Aguilar et al., 2004).

### Library construction

Total RNA was extracted from root tissue using TRIZOL (Thermo Fisher) and treated with RNase-free DNase (Promega). RNA quality was evaluated by capillary gel electrophoresis in an Agilent 2100 Bioanalyzer using the RNA 6000 Nano kit (Agilent). Libraries were prepared using 3 µg of total RNA and the Illumina TruSeq RNA Sample Preparation kit v2 following provider’s instructions. Superscript II (Invitrogen) reverse transcriptase was used for cDNA synthesis. PCR fragments were purified using AMPure XP beads (Beckman Coulter Genomics). Size and concentration of the synthesized DNA fragments were verified in an Agilent 2100 Bioanalyzer using the DNA-1000 kit (Agilent). Libraries were equalized and multiplexed following Illumina recommendations for barcode compatibility and sequenced in an Illumina HiSeq4000 at the HTS service of the University of California Davis DNA Technologies Core. The number and quality of the 90-mer single end reads obtained for each condition are listed in Supplementary Table S1. Raw data were deposited at Gene Expression Omnibus database under the accession GSE155568.

### Data analysis

Illumina reads were aligned to the *P. vulgaris* genome using Tophat 2^18^. Data analyses were performed essentially as previously reported^34^. Minimum and maximum intron lengths were set as 60 and 6000 and the options own junction usage and indel search were activated. Reads were aligned using the v2.1 of the *Phaseolus vulgaris* genome obtained from the Andean G19833 accession.

Transcript assembling was performed using Cufflinks and differentially expressed genes were identified with Cuffdiff^37,38^ using quartile normalization, BIAS correction and multi-read correct options. A Cuffmerge analysis including Cufflink results from each sample was used to quantify the expression of genes and transcripts^38^. All possible pairwise comparisons were run and genes with an expression value higher than 1 fragment per kilobase per million (FPKM), with a fold change ≥ 2 and a *p* value below 0.05 were considered as differentially expressed (DEGs) (Supplementary Tables S4 and S5).

Non-coding RNAs were identified using the Coding Potential Calculator 2 algorithm^28^. Non-coding genes were defined as those that produce RNAs longer than 200 base pair, does not have an ORF of more than 300 bases, have a Coding probability lower than 0.5 and a HMMER (http://hmmer.org) alignment full sequence E value higher than 0.01.

Principal Component Analysis (PCA) was performed with the PAST3 software (PAleontological Statistics version 4.03, https://www.nhm.uio.no/english/research/infrastructure/past/)^39^. The log_2_ value expression normalized by the control (YEM sample) was used for the correlation method. Venn diagrams were obtained using the tools available in the bioinformatic webpage of the Ghent University (http://bioinformatics.psb.ugent.be/webtools/venn).

To obtain promoter sequences of DEGs, the regions from − 1500 to + 100 bp respect to the translational start site were extracted from the fasta file containing the genome of the BAT93 variety of *P. vulgaris*^26^ using bedtools v2.29 suite (https://bedtools.readthedocs.io/). As the assembling of the genome is not fully completed, sequences shorter than 300 bp were filtered. Promoter regions were analyzed with the tool Regulation prediction, available at the Plant Transcriptional Regulatory Map webpage (http://plantregmap.gao-lab.org/), using default parameters.

### Reverse transcription and quantitative PCR

Expression analysis by reverse transcription followed by quantitative PCR (RT-qPCR) was performed essentially as previously described^14^. For each primer pair, the presence of a unique PCR product of the expected size was verified in ethidium bromide–stained agarose gels. Absence of contaminant genomic DNA was confirmed in reactions with DNase-treated RNA as template. Amplification of common bean *Eukaryotic Elongation factor 1 α* (*EF-1α*) transcript was used to normalize the amount of cDNA template. Three biological replicates independent of those used for library construction were used for each condition. Primers used are listed in Supplementary Table S10.

## Supplementary Information

Supplementary Legends.

Supplementary Table S1.

Supplementary Table S2.

Supplementary Table S3.

Supplementary Table S4.

Supplementary Table S5.

Supplementary Table S6.

Supplementary Table S7.

Supplementary Table S8.

Supplementary Table S9.

Supplementary Table S10.

## Data Availability

All the experiments performed in this study complied with local and national regulations.
