# Supplementary material for: Transcriptomic analysis of Mesoamerican and Andean Phaseolus vulgaris accessions revealed mRNAs and lncRNAs associated with strain selectivity during symbiosis
Source: Sci Rep. 2022 Feb 16;12:2614. doi: 10.1038/s41598-022-06566-0 (PMC8850587; doi:10.1038/s41598-022-06566-0)
Supplement: Supplementary file 1 — Supplementary Legends. [file 41598_2022_6566_MOESM1_ESM.doc]

**Supplementary Tables**

**Table S1:** Number of reads and quality of RNA-seq libraries. Illumina reads were aligned to the P. vulgaris genome using Tophat 2. Coverage was calculated using Plot coverage, available in the Galaxy Platform (https://usegalaxy.org/). The assembly includes 473 Mb of the 587-Mb estimated in the P. vulgaris genome.

**Table S2.** Normalized expression values obtained for all NAG12 genes. Mean expression values obtained for all NAG12 genes (expression values per replicate are provided in Sheet2). Values are expressed in fragments per kilobase per million reads (FPKM) for each gene. Expression values were calculated from samples mock-inoculated with culture media (YEM) or inoculated with the R. etli strains SC15, CE3, 55N1 and 124N1. The identification number of each gene (Gene ID), the transcription start site/s (TSS), the location in the genome (Locus) and functional annotation are provided for each gene.

**Table S3.** Normalized expression values obtained for all Alubia genes. Mean expression values obtained for all Alubia genes (expression values per replicate are provided in Sheet2). Values are expressed in fragments per kilobase per million reads (FPKM) for each gene. Expression values were calculated from samples mock-inoculated with culture media (YEM) or inoculated with the R. etli strains SC15, CE3, 55N1 and 124N1. The identification number of each gene (Gene ID), the transcription start site/s (TSS), the location in the genome (Locus) and functional annotation are provided for each gene.

**Table S4. Differential expression testing for all NAG12 genes in response to each *R. etli* strain.** Systematic pairwise comparisons for each gene are shown. Expression values are expressed in fragments per kilobase per million reads (FPKM).

**Table S5. Differential expression testing for all NAG12 genes in response to each *R. etli* strain.** Systematic pairwise comparisons for each gene are shown. Expression values are expressed in fragments per kilobase per million reads (FPKM).

**Table S6.** Classification of differentially expressed genes (DEGs) into functional categories. Common and strain specific DEGs upon inoculation with *R. etli* strains are shown. The number of DEGs, the gene identification number (Id), the annotation and functional classification are shown for each strain or group of strains.

**Table S7.** Differential expression testing of nodulation signaling pathway genes in NAG12 and Alubia in response to each *R. etli* strain. The gene identification number (ID) and the significance of differential expression (YES or NOT) as compared to the control sample (YEM) is shown for each gene involved in the nodulation signaling pathway.

**Table S8. Regulatory *cis*-elements present in the promoter regions of DEGs.** Number of *cis*-elements in the different groups of NAG12 DEGs present in the promoter regions between the -1500 and the +100 of the translation start site (Values for Alubia are provided in sheet 2).

**Table S9. lncRNAs identified as DEGs in all pairwise comparisons.** The number of differentially expressed lncRNAs, the identification number of each novel transcript, gene id (for annotated genes), the transcription start site/s (TSS_id), the location in the genome (Locus) and the expression values expressed in fragments per kilobase per million reads (FPKM) are shown for each strain or group of strains. Each group defined in figure 9 is shown in a different sheet of the table.

**Table S10. Primer sequences.** Sequences of the primers used to quantify the expression levels of the indicated genes by qRT-PCR. EF1-α was used as reference gene
